# Knowledge and Attitudes of Healthcare Workers towards Antibiotic Use and Antimicrobial Resistance in Two Major Tertiary Hospitals in Western Greece

**DOI:** 10.3390/antibiotics12111583

**Published:** 2023-11-02

**Authors:** Maria Lagadinou, Elpida Tsami, Apostolos Deligakis, Themistoklis Paraskevas, Christos Michailides, Dimitrios Velissaris, Despoina Gkentzi, Markos Marangos

**Affiliations:** 1 Department of Internal Medicine, University General Hospital of Patras, 26504 Patras, Achaia, Greece; themispara@hotmail.com (T.P.); christos.mich1@gmail.com (C.M.); dvelissaris@upatras.gr (D.V.); marangos@upatras.gr (M.M.); 2 Department of Medical School, University of Patras, 26504 Greece, Achaia, Greece; gkentzid@upatras.gr; 3 Department of Surgery, “Saint Andrew” General Hospital of Patras, 26334 Patras, Achaia, Greece; tsamielpis@hotmail.com (E.T.); adeligakis@yahoo.gr (A.D.); 4 Department of Pediatrics, University General Hospital of Patras, 26504 Patras, Achaia, Greece; 5 Department of Infectious Diseases, University General Hospital of Patras, 26504 Patras, Achaia, Greece

**Keywords:** antibiotics, antibiotic use, resistance, healthcare workers

## Abstract

Antibiotic resistance (ABR) and antimicrobial stewardship arethe two sides of the same coin that constitute a public health hydra. This study aimed to assessthe knowledge and attitude of healthcare workers (HCWs), on antibiotic use and antimicrobial resistance in Western Greece. A total of 200 healthcare workers (doctors, nurses, and others) from the two largest tertiary hospitals in Western Greece were included in our survey. HCWs seem not to decide based on patient opinion in order to prescribe antibiotics. Approximately 97% of them are aware of their main adverse effects. Remarkably, 25% of respondents prescribe antibiotics due to diagnostic uncertainty, and 32.5% of them prescribe antibiotics based on their experience. HCWs statedthat they do not report adverse effects often. Inappropriate antibiotic prescriptions were mentioned as the main reason for bacterial resistance to antimicrobials. Monitoring the patient’s treatment progress, using electronic prescriptions, and adhering to international guidelines were suggested as solutions to the problem. Post Hoc analysis showed that nursing staff apply to the national guidelines (p: 0.011) and use electronic prescriptions (p: 0.003) less often compared to consultants, doctor directors, and trainees. The findings of our survey may be useful for the development of future national education programs and interventions thatmay improve healthcare workers’ knowledge and ability to manage antibiotics.

## 1. Introduction

Antibiotics’ rampant use and inappropriate prescriptions lead to antimicrobial resistance. According to WHO, 20–50% of the antibiotics distributed to primary care are misused [1]. At present, antibiotic resistance (ABR) is recognized as one of the most serious threats worldwide, leading to severe unfavorable outcomes in infected patients [2,3]. A total of 700,000 deaths are caused by infections with antibiotic-resistant microorganisms; thus, this threat should be taken into consideration [4]. Healthcare workers are directly involved in the prescribing and use of antibiotics. Generally, they might be affected by patients’ inappropriate requests and may prescribe inappropriately [2]. Possible causes of that inappropriateness are a lack of knowledge about their pharmacology and treatment evidence [4]. Recent studies have reported that antibiotic resistance spread was rapid in areas where HCWs were unaware of the problem [5].The European Centre for Disease Prevention and Control (ECDC) organizes programs to assess HCWs baseline knowledge, attitudes, and behaviors surrounding antibiotics in 2019 [4].

Greece has one of the highest rates of antibiotic consumption and resistance among European countries. The link between antimicrobial consumption and resistance is well-known [6]. Even though antibiotics are medications under limitation, self-medication is common in Greece. The major source of self-medication in Greece is acquisition from pharmacies without a prescription and over-the-counter consumption of left-over antibiotics at home. Over-the-counter consumption is a worldwide problem that directly leads to antimicrobial resistance. Until 2008, in Greece, amoxicillin-clavulanate and ciprofloxacin could be purchased without a prescription [7].

The implementation of measures to restrict the use of certain antibiotics has been proven to reduce inappropriate antimicrobial administration. Following the introduction of electronic prescribing and Antibiotic Hardstop, a significant reduction has been observed in the days of therapy and length of therapy for antimicrobial use without a significant increase in adverse events [8].

The aim of the present study was to assess the knowledge and attitude of healthcare workers (HCWs), regarding antibiotic use, side effects, overprescription, and consequently antimicrobial resistance. The results of this study may help identify those HCWs with knowledge gaps and assist in the design of future educational interventions that will enable more rational antimicrobial prescribing.

## 2. Materials and Methods

### 2.1. Study Design, Participants, Questionnaire

This is a cross-sectional observational study that was conducted in the two largest tertiary hospitals (the General University Hospital of Patras and the General Hospital of Patras, “Agios Andreas”)in Western Greece, based on a scripted questionnaire. The questionnaire was randomly distributed to healthcare workers, including residents, consultants, nurses, technicians, and others. Healthcare workers were invited to participate in our survey during a period of 3 months (May to July 2023).An overview of this study objectives was provided to potential participants.

The questionnaire was pre-developed by independent members of our research team based on pertinent current literature [2,9,10]. In order to ensure clarity, content validity, and internal consistency, there was a short pilot phase when 20 healthcare providers answered and commented on the survey. Following the review of the issues raised by the pilot study participants, the questionnaire was adjusted accordingly by the research team to its final form.

The questionnaire was written in Greek and consisted of three parts. Part A included six questions regarding demographic characteristics (age, gender, job experience, work department, and medical specialty). Part B included five questions regarding knowledge of antibiotic use (factors affecting antibiotic prescription and administration, side effects related to antibiotics’ use, and side effects report). Part C included seven questions regarding knowledge and attitude towards antibiotic resistance.

This study was conducted in line with the Ethics Committee of our University (Approval Number 40136/13.07.2023), the guidelines of the Declaration of Helsinki, and STROBE guidelines.

### 2.2. Statistical Analysis

Statistical analysis was conducted using IBM SPSS Statistics, version 28. The level of significance was set to 0.05. Descriptive statistics were used to summarize all the data on demographic characteristics, knowledge, and attitudes regarding antibiotics, antibiotics’ use, and resistance. Post Hoc analysis was used to determine any statistically significant difference between the parameters compared. Comparisons between pairs of groups were performed using the simplest post hoc analysis, the Bonferroni test.

## 3. Results

### 3.1. Demographic Characteristics

A total of 200questionnaires, out of 520 randomly administered, were answered and included in this study (response rate: 38.5%). Among the respondents, 74.5% (n = 149) were females and 25.5% (n = 51) were males. Socio-demographic and professional characteristics (age, sex, medical specialty, work experience) of the participants are presented in Table 1.

### 3.2. Healthcare Workers’ General Knowledge and Attitude towards Antibiotic Use

**Figure 1:**
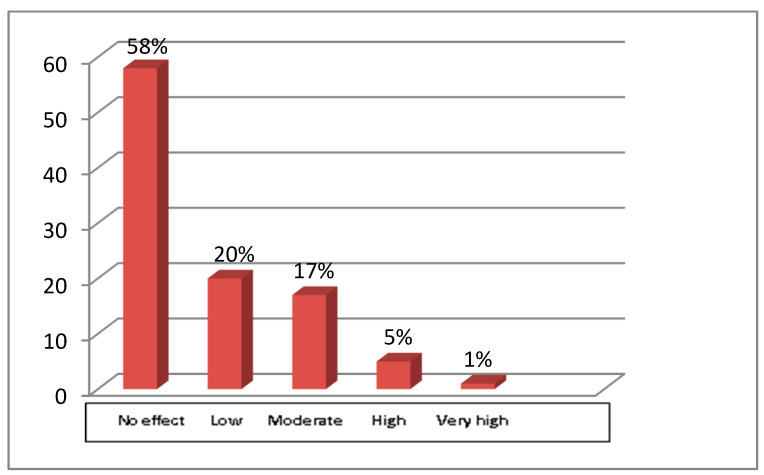
The present figure presents the extent to which patients’ requirements affect HCWs decisions regarding the administration of antibiotics. The x axis represents the extent to which HCWs are influenced by patients’ requests, and the y axis represents the percentage of HCWs.

General knowledge regarding antibiotic use was confirmed in many questions (i.e., factors resulting in unnecessary use of antibiotics, side effects related to antibiotics’ use, etc.). In terms of antibiotics’ use, most of the participants (32.5%) answered that they often prescribe antibiotic regimens based on their experience and 25% due to diagnostic uncertainty. Patients’ requests for involvement in HCWs decisions to prescribe antibiotics are shown in Figure 1.

Almost all participants were aware of common antimicrobial regimen side effects, including gastrointestinal tract symptoms and allergic reactions, as shown in Table 2.

Table 3 summarizes the way HCWs report side effects. Interestingly, several participants (n = 79, 39.5%) stated that they informed their director about adverse events instead of completing the National Drug Agency’s yellow card or communicating with the National Drug Agency.

Curiously, several participants stated that they have never reported antibiotic side effects due to an extensive workload (n = 69, 34.5%), or even because they degraded them (n = 65, 32.5%). Another frequent reason was the lack of knowledge regarding the obligation to declare adverse side effects (n = 49, 24.5%). Moreover, post hoc analysis showed that trainees had statistically significantly less knowledge and skills to recognize adverse reactions after the administration of antibiotics compared to consultants and directors (p = 0.001).

### 3.3. Healthcare Workers’ General Knowledge and Attitude towards Antibiotic Resistance

**Figure 2:**
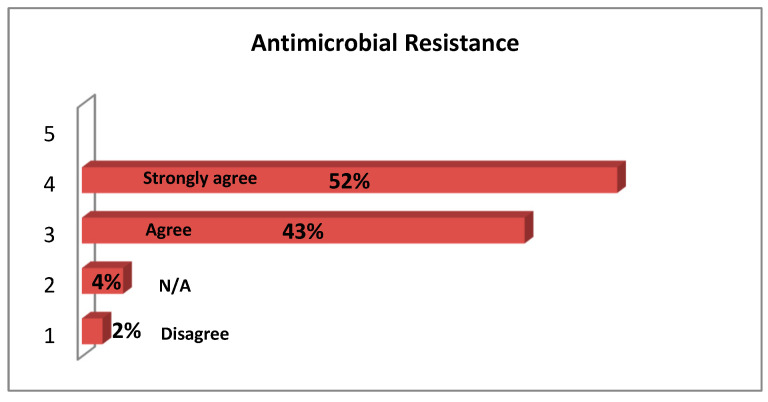
Attitudes of Healthcare workers regarding whether Antimicrobial Resistance is a major Public Health problem in Greece. N/A: not applicable.

Almost all of the participants (97%) stated that antibiotic resistance is a serious public health issue in Greece. (Figure 2).

The most frequent explanation (64.5%) for increased antimicrobial resistance in Greece was that antibiotics are used inappropriately by HCWs. Overprescription (61.5 %), and over-the-counter consumption (55.5%) were two other frequent explanations (Table 4).

In order to mitigate the consequences of rampant use of antibiotics, HCWs responded that they try to adhere to local guidelines, use e-prescription, conform to current evidence, and track patients to observe their outcomes (Table 5).

Post Hoc analysis showed that nursing staff have less adherence to guidelines compared to consultants, directors, and trainees (p = 0.011).The use of electronic prescription systems among doctors has strong statistical significance compared to nursing stuff (p: 0.003). No statistically significant differences were found regarding continuing education and monitoring of patients care (p = 0.052 and p = 0.299, respectively) between nurses and doctors (specialized, consultant, or director).

## 4. Discussion

Antibiotic resistance and antibiotic stewardship are crucial factors that undermine the quality of healthcare systems. This is the first study in Western Greece that underlines the urge for proper HCWs education and strict application of rules and guidelines.

Our survey investigated healthcare workers’ knowledge of antibiotic use and antibiotic resistance, as HCWs are the key stakeholders in the prevention and control of antibiotic resistance. Apparently, HCWs knowledge of antimicrobial regimens’ mechanisms of action and resistance mechanisms would prevent further increases in antimicrobial resistance [11]. This study showed that although healthcare workers are aware of the potential threat of antibiotic resistance, their knowledge does not seem to be sufficient for the appropriate use of antimicrobial agents. Antimicrobial stewardship programs (ASPs) could enhance HCW comprehension and help address this issue [12].

Unfortunately, 32.5% of the participants answered that they often prescribe antibiotic treatment based on their experience and 25% due to diagnostic uncertainty. Similarly, Christensen I. et al. reported that even in Norway, where antimicrobial resistance rates are among the lowest in Europe, one of the main reasons physicians prescribed antibiotics was clinical uncertainty [11]. Moreover, two systematic reviews reported that the cause of the inappropriate use of antibiotics is the fear of patients’ unfavorable outcomes [11,13,14]. The latter reinforces the need to develop rapid point-of-care diagnostic and prognostic tests that would remove this uncertainty and thereby enable more appropriate prescribing [15].

This study showed poor knowledge and practice regarding antibiotic use. Most participants answered that they inform their director about side effects related to antibiotics’ use instead of writing the appropriate yellow card. This is due to a lack of both knowledge and experience. Such findings reflect the lack of efficacious educational programs. HCWs, especially nurses and trainees, need to extend their knowledge regarding antibiotic use [9]. With all the aforementioned matters, the need to enhance young HCWs education regarding antibiotic use and antimicrobial resistance, even during their studies, is urgent [16]. As far as time organization is concerned, curricula studies have shown that the early introduction of antimicrobial resistance teaching as well as repetitive and enriched training on antibiotic resistance, prescribing, and communication skills would lead to a more comprehensive understanding of the challenges and complexities of antibiotics’ management [16,17].

Nurses dramatize a crucial role in efforts to address this worldwide public health problem. Although in Greece nurses do not prescribe antibiotics, this study aims to evaluate their knowledge regarding national guidelines and the electronic prescription system. As anticipated, nurses performed insufficiently on practices related to antibiotic use (apply the national guidelines, use the electronic prescription). These findings are in line with a study by Farley et al., which revealed disparity among nurses concerning the awareness and understanding of optimal antibiotic prescribing practices [10]. However, some studies reported that nurses were familiar with antibiotic use [18,19]. Explicitly, Jayaweerasingham et al. reported that nurses’ knowledge among their study cohort appeared to be adequate, even though some misbeliefs were objected to [18].

Almost all the participants (97%) in the current study agreed with the statement that antimicrobial resistance is a significant public health issue in Greece. Similarly, Firouzabadi et al. and Mahmoudi et al. reported that antimicrobial resistance was considered a crucial problem by 73.8% of the participants [12]. Remarkably, in a study by Garcia et al., most of the participants considered AMR a threat in primary care rather than in the hospital setting [20].

As most of the participants mentioned unrestricted prescription and administration of antibiotics as the main contributing factors to antimicrobial resistance, 46% of them agreed that monitoring patients’ treatment progress can reduce antibiotic resistance. Further, 43% of them agreed that the use of electronic prescriptions can reduce antibiotic resistance. Cotta MO et al. data were inconsistent with our findings, demonstrating that limiting antibiotic prescription by an approval process, such as electronic prescribing, was the least approved intervention [21]. Finally, in our study, 32% of the participants agreed that adherence to international guidelines can reduce antibiotic resistance. Similarly, in a study by Srinivasan A. et al., 97% of the participants stated that the correct selection of antibiotics can help reduce AMR [22]. Even since 2002, the CDC has included several measures pertaining to improved antimicrobial use in its “Campaign to Prevent Antimicrobial Resistance in Health-Care Settings” [23].

Our study, as a real-world study, has some limitations. At first, it was a two-center study with a small number of participants. Secondly, participation in our study was voluntary; therefore, HCWs feeling uncomfortable may have deliberately decided not to participate, causing selection bias. Another limitation is that participants may give desirable answers rather than their actual beliefs. For those reasons, we cannot generalize these findings.

However, the main strength of our study is that we included qualitative parameters, which helped us explore the ideas prevalent among this study population that would not have been revealed in a quantitative study.

## 5. Conclusions

In our study, a knowledge deficit was observed in some aspects of antimicrobial use. The findings of our survey may be useful for the development of future educational programs and interventions that focus specifically on promoting behaviors that lead to prudent prescription, dispensing, and administration of antibiotics. Furthermore, they highlight the need to continue to raise awareness about appropriate and correct use of antibiotics and antibiotic resistance and to enhance healthcare workers’ engagement in addressing these issues. Finally, coordinated efforts to implement new policies, renew research efforts, and pursue steps to manage the crisis are greatly needed.

## Figures and Tables

**Table 1:** Socio-demographic and professional characteristics of this study population.

Gender	F	%
Male	51	25.5
Female	149	74.5
Age(years)		
22–30	49	24.5
31–40	52	26.0
41–50	76	38.0
51–60	21	10.5
>61	2	1.0
Medical Specialty		
Internist	19	9.5
General Practitioner	8	4.0
Nurse	111	55.5
Infectious Diseases	1	0.5
Cardiologist	5	2.5
Surgeon	18	9.0
Other	38	19.0
Work Department		
Director	5	2.5
Consultant	38	19.0
Trainees	26	13.0
Nurses	110	55.0
other	21	10.5
Work Experience(years)		
0–3	37	18.5
4–7	41	20.5
8–11	23	11.5
12–19	47	23.5
>20	52	26.0

**Table 2:** Side effects associated with antibiotic use. This table summarizes perceived knowledge related to side effects associated with taking antibiotics. Numbers show the number of participants who gave a specific answer.

Frequency of Side Effects					
Side Effect	Zero	Low	Moderate	High	Very High
Nausea	1 0.5%	23 11.5%	71 35.5%	70 34.4%	34 17.0%
Vomiting	7 3.5%	26 13.0%	72 36.0%	62 31.0%	33 16.5%
Diarrhea	1 0.5%	10 5.0%	28 14.0%	86 43.0%	75 37.5%
Rash	3 1.5%	20 10.0%	60 30.0%	64 32.0%	53 26.5%
Abdominal Pain	8 4.0%	33 16.5%	63 31.5%	65 32.5%	31 15.5%
Allergies	3 1.5%	24 12.0%	45 22.5%	63 31.5%	65 32.5%

**Table 3:** Reporting adverse events. This table summarizes the answers given regarding the report of side effects. Most participants answered that they just inform their director.

	N	%
Communication with National Drug Agency	19	9.5%
Completion of National Drug Agency’s yellow card	57	28.5%
Contact with the responsible Pharmaceutical Company	9	4.5%
Inform the Director	79	39.5%
Ι have not noticed or reported any side effects	19	9.5%
Although I have noticed, I have not reported any side effects	17	8.5%

**Table 4:** Main reasons for the increase in antibiotic resistance. The numbers show the absolute number of participants who gave a specific answer.

Frequency					
	Zero	Low	Moderate	High	Very High
The long duration of antimicrobial therapy	4 2.0%	3 1.5%	40 20.0%	82 41.0%	71 35.5%
Poor hand hygiene	8 4.0%	14 7.0%	33 16.5%	53 26.5%	92 46.0%
The arbitrary use of antibiotics	1 0.5%	3 1.5%	18 9.0%	49 24.5%	129 64.5%
Over the counter consumption	6 3.0%	3 1.5%	20 10.0%	60 30.0%	111 55.5%
Aggressive prescribing	1 0.5%	4 2.0%	13 6.5%	59 29.5%	123 61.5%
The administration of broad spectrum antibiotics	15 7.5%	13 6.5	35 17.5	69 34.5%	68 34.0%

**Table 5:** Summarizes the frequency with which some measures were suggested by participants for restricting uncontrolled prescribing. The number shows the absolute number of participants who gave a specific answer.

	No Often	Quite Often	Sometimes	Often	Very Often
Adherence to international guidelines	6 3.0%	11 5.5%	56 28.0%	63 31.5%	64 32.0%
Use of e-prescription	12 5.5%	16 8.0%	27 13.5%	59 29.5%	86 43.0%
Continuing education on antibiotic evolution and prescription	8 4.0%	13 6.5%	32 16.0%	82 41.0%	65 32.5%
Monitoring of patient’s treatment progress	2 1.0%	16 8.0%	27 13.5%	63 31.5%	92 46.0%

## Data Availability

The datasets generated and analyzed during the current study are available upon reasonable request to the corresponding author.
